# Publisher Correction: Anderson light localization in biological nanostructures of native silk

**DOI:** 10.1038/s41467-018-03595-0

**Published:** 2018-03-19

**Authors:** Seung Ho Choi, Seong-Wan Kim, Zahyun Ku, Michelle A. Visbal-Onufrak, Seong-Ryul Kim, Kwang-Ho Choi, Hakseok Ko, Wonshik Choi, Augustine M. Urbas, Tae-Won Goo, Young L. Kim

**Affiliations:** 1 Weldon School of Biomedical Engineering, Purdue University, West Lafayette, IN 47907 USA; 2 Department of Agricultural Biology, National Institute of Agricultural Sciences, Rural Development Administration, Wanju, 55365 Republic of Korea; 3 Materials and Manufacturing Directorate, Air Force Research Laboratory, Wright-Patterson Air Force Base, OH 45433 USA; 4 Center for Molecular Spectroscopy and Dynamics, Institute for Basic Science, Seoul, 02841 Republic of Korea; 5 Department of Physics, Korea University, Seoul, 02841 Republic of Korea; 6 Department of Biochemistry, School of Medicine, Dongguk University, Gyeongju, 38066 Republic of Korea; 7 Regenstrief Center for Healthcare Engineering, Purdue University, West Lafayette, IN 47907 USA; 8 Purdue Quantum Center, Purdue University, West Lafayette, IN 47907 USA

Correction to: *Nature Communications* 10.1038/s41467-017-02500-5, published online 31 January 2018

The original PDF version of this Article contained errors in Equations 1 and 2. Both equations omitted all Γ terms, and incorrectly read:

1 $$I_m\left( {{\mathbf{r}},E_{{\mathrm{ex}}},\omega } \right) = \mathop {\sum }\limits_{h = 1}^H a_h({\mathbf{r}},E_{{\mathrm{ex}}})\frac{{\,_h({\mathbf{r}},E_{{\mathrm{ex}}})/2}}{{i\left( {\omega - \omega _h\left( {{\mathbf{r}},E_{{\mathrm{ex}}}} \right)} \right) + \,_h({\mathbf{r}},E_{{\mathrm{ex}}})/2}}$$

2 $$\min \,F = \sqrt {\frac{1}{S}\mathop {\sum }\limits_{S = 1}^S ||I_e\left( {\omega _s} \right) - I_m(\omega _s,a_h, \omega _h,_h)||^2}$$

The correct form of Equations 1 and 2 are:

1 $$I_m\left( {{\mathbf{r}},E_{{\mathrm{ex}}},\omega } \right) = \mathop {\sum }\limits_{h = 1}^H a_h({\mathbf{r}},E_{{\mathrm{ex}}})\frac{{\Gamma _h({\mathbf{r}},E_{{\mathrm{ex}}})/2}}{{i\left( {\omega - \omega _h\left( {{\mathbf{r}},E_{{\mathrm{ex}}}} \right)} \right) + \Gamma _h({\mathbf{r}},E_{{\mathrm{ex}}})/2}}$$

2 $$\min \,F = \sqrt {\frac{1}{S}\mathop {\sum }\limits_{S = 1}^S || I_e\left( {\omega _s} \right) - I_m(\omega _s,a_h,\omega _h,\Gamma _h) || ^2}$$

This has been corrected in the PDF version of the Article. The HTML version was correct from the time of publication.

