# Supplementary material for: Anderson light localization in biological nanostructures of native silk
Source: Nat Commun. 2018 Jan 31;9:452. doi: 10.1038/s41467-017-02500-5 (PMC5792459; doi:10.1038/s41467-017-02500-5)
Supplement: Supplementary file 1 — Supplementary Information(PDF 2622 kb) [file 41467_2017_2500_MOESM1_ESM.pdf]

Supplementary Information

Supplementary Methods

1. Imaging ultrastructure of silk

Scanning electron microscopy (SEM) Using SEM, we imaged the surface of freeze fractured fibroin filaments to investigate the morphology of nanofibrils, interfibrillar voids, silk fibres, and cocoons. For freeze fracture, the specimens were dipped in liquid nitrogen for 30 minutes and were quickly fractured with a sharp edge of a clean razor blade. The polymeric nature of silk proteins (i.e. fibroin), such as low atomic weight, low density protein, and rapid degradation to electron beam, often limits the use of electron microscopy[1,2]. Thus, the specimens were coated with platinum (Pt) for 60 seconds using a Cressington sputter coater (Ted Pella, Inc). The specimens were imaged using a NOVA nanoSEM field emission scanning electron microscope (FEI, Co) with an Everhart-Thornley detector or a high-resolution through-the-lens detector (TLD), at an accelerating voltage of 5kV. The SEM micrographs clearly show numerous individual nanofibrils separated by voids (Fig. 1b, c).

Transmission electron microscopy (TEM) Using TEM, we imaged the cross-section of nanofibrillar structures in silk fibres to characterize their sizes and interfibrillar spaces. Typically, TEM is more challenging than SEM for imaging the ultrastructure of silk fibres[1-3]: (i) For polymeric silk proteins, staining methods are not well established. (ii) Silk proteins are subject to rapid degradation upon electron beam irradiation. (iii) Ultrathin sections with proper orientations (i.e. transverse and longitudinal cross sections) of nanofibrils are difficult. Thus, we made use of a two-step metal staining method for enhancing the image contrast of nanofibrils. The silk fibre specimens were fixed overnight with osmium tetroxide (OsO_4) vapour and then were infiltrated with Spurr's resin. After polymerization, the specimens were sectioned with a thickness of 100 nm. The sections

underwent post staining with uranyl acetate in 70% methanol in five minutes and lead citrate in three minutes. The specimens were examined using a Tecnai T20 transmission electron microscope at 200 kV with a lanthanum hexaboride filament (FEI, Co). Micrographs were captured using a Gatan camera (Gatan, Inc). The TEM micrographs show areas of nanofibrils, sericin, and interfibrillar voids (Fig. 1d−f and Supplementary Figure 1a−c). Nanofibrils were delineated from the density contrast resulting from more electrically dense nanofibrils and less dense voids, due to metal staining. The distributions of nanofibril sizes and interfibrillar spaces were extracted by the binary images that were post-processed from the TEM micrographs (Supplementary Figure 1a).

Molecular imaging of nanofibrils As a S158A variant of mKate, mKate2 (far-red fluorescent protein) is more pH-stable and has higher brightness at physiological pH[4] (Supplementary Figure 2e). To express mKate2 in nanofibrils of silk filaments, we genetically engineered domesticated silkworms (*Bombyx mori* bivoltine strain and Supplementary Figure 2a), by means of silkworm transgenesis using germline transformation[5-7]. The transformation vector p3xP3-EGFP-pFibH-mKate2 was constructed as the *piggyBac*-derived vector and was injected with a helper vector pHA3PIG into pre-blastoderm embryos[8] (Supplementary Figure 2b). The mKate2 gene was fused with N-terminal and C-terminal domains of the fibroin heavy chain promoter (pFibH), confirmed by high-performance liquid chromatography with tandem mass spectrometry (LC-MS/MS) (Supplementary Figure 2c, d). The 3xP3-EGFP system allowed us for screening a large number of G1 broods to identify transgenic silkworms[9]. The hatched larvae and silkworms were reared at 25°C and were fed on mulberry leaves as well as an artificial diet. As a result, red fluorescence signals appear in the silk gland (Supplementary Figure 2f), the silk cocoons (Supplementary Figure 2g), and the nanofibrils (Supplementary Figure 3).

2. Calculation of the localization length of light ξ

The localization length of light ξ is an intrinsic ensemble averaged decay length of localized modes, which is a statistical property in a finite system. Thus, it is important to investigate numerous structures of statistical equivalence. An ensemble average transmittance $< T >$ over 100 different realizations of nanofibrillar structures were computed at different system lengths L (Supplementary Figure 4a, b)[10-12]. The logarithmic decay was stabilized beyond $L > 5$ μm and converged to a single exponential:

$$< \ln T > \approx -L / 2\xi \,, \tag{1}$$

which returns $\xi = 4.5$ μm.

The field shape (i.e. spatial extension) is an alternative route to estimate ξ (Supplementary Figure 4c, d)[12-14]. The spread of wavefunction was quantified by an inverse participation ratio (IPR):

$$\mathrm{IPR}\big(E(x)\big) = \frac{\int \mathrm{d}x \left|E(x)\right|^4}{\left|\int \mathrm{d}x \left|E(x)\right|^2\right|^2} \,. \tag{2}$$

This length parameter relates ξ such that $1/\mathrm{IPR}(E(x)) = 2\xi$, which enables to model an exponential field peak of a localized mode:

$$E(x) \sim \frac{e^{-|x|/2\xi}}{\sqrt{2\xi}} \,. \tag{3}$$

In Fig. 2a, the field patterns are in good agreement with the expression of Supplementary Eq. (3), when ξ is substituted with their spreads of 1/IPR.

3. Measurements of the mean free path length of light in silk

Describing the scattering properties of biological and natural tissues, the scattering mean free path length of light l_s is related to the transport mean free path length l_t such that[15-18]:

$$l_s = \frac{1}{\mu_s} = (1 - \langle \cos\theta \rangle) l_t \, , \qquad (4)$$

where $<\cos\theta>$ is the scattering anisotropy factor of a single scattering event, μ_s is the scattering coefficient, and l_t is the transport mean free path length, which is measurable by coherent backscattering of light[19-22]. For the white silk cocoon specimen, the angular profile of coherent backscattering (CBS) (Supplementary Figure 5b) returned $l_t \approx 16 \pm 0.2$ μm. Typically, biological and natural tissue has a nonzero scattering anisotropy factor (i.e. $l_s \neq l_t$)[23-25], while the scattering anisotropy factor of typical powder samples is zero (i.e. $l_s = l_t$). This difference originates from the scattering phase function $p(\mathbf{s},\mathbf{s}')$, which is a probability of scattering into the direction **s'** from **s**, leading to interpretation of light propagation from a particle standpoint[23]:

$$\frac{\partial R(\mathbf{r},\mathbf{s})}{\partial s} = -(\mu_a + \mu_s) R(\mathbf{r},\mathbf{s}) + \frac{\mu_s}{4\pi} \int_{4\pi} R(\mathbf{r},\mathbf{s}') p(\mathbf{s},\mathbf{s}') \; \mathrm{d}\Omega' \, , \qquad (5)$$

where $R(\mathbf{r},\mathbf{s})$ is the radiance at position **r** traveling in the direction **s**, $d\Omega'$ is the unit solid angle about **s**', and μ_a is the absorption coefficient. From $p(\mathbf{s},\mathbf{s}')$, the directionality of a single scattering event can be quantified, assuming symmetric scattering around the incidence direction $p(\mathbf{s},\mathbf{s}') = p(\theta)$:

$$\langle \cos\theta \rangle = \int_0^{\pi} p(\theta) \cos\theta \, 2\pi \sin\theta \; \mathrm{d}\theta \, , \qquad (6)$$

where θ is the angle between **s'** and **s**. To measure $<\cos\theta>$ experimentally, we conducted an integrating sphere measurement incorporating an inverse adding-doubling computation[26]. For the white silk cocoon, the scattering anisotropy factor of an effective single scattering event $<\cos\theta> = 0.78$, returning $l_s = (1 - <\cos\theta>) \times 16$ μm = 3.5 μm. We note that the current CBS measurements of l_t and l_s are the bulk scattering properties averaged over a large illumination area (3 mm × 3 mm). These values cannot appropriately reflect the event of light localization that occasionally occurs in a small area over a single filament (Fig. 4f, g).

To account for the uncertainty on l_t due to the refractive index mismatch between the silk sample and the surrounding medium, we cross-validated the value of l_t with extinction experiments using an integrating sphere[26]. This method incorporates the solution of the radiative transport equation that models an additional layer for each mismatched boundary and thus it is technically free from the issue about the surface/internal reflection at the boundary. From the extinction experiments, the measured l_s is 3.48 μm and the deduced l_t from the anisotropy factor is 15.8 μm at 543.5 nm. Compared with the CBS measurement, the reduction in l_t from this alternative measurement is only 1.2%.

Using $\theta_{\mathrm{FWHM}}^{-1} = \frac{2}{3}\left(1 + \frac{1+R_{\mathrm{s}}}{1-R_{\mathrm{s}}}\right) k l_{\mathrm{t}}$, one can also predict a CBS angular width taking the surface reflectivity R_s (specular reflection)[27] into account. The refractive index of silk protein (i.e. fibroin) n_{silk} = 1.6 and the volume fraction of nanofibrils f = 0.3 result in an effective refractive index of 1.16. In this case, the correction factor $1/(1 - R_s)$ = 0.54% yields a corrected CBS value of l_t to be 15.9 μm, which is in good agreement with the result from the extinction experiments. Typically, the surface reflectivity makes large uncertainty when CBS is applied to high-refractive-index specimens such as TiO_2[28,29]. However, silk does not have such a high refractive index to induce a significant index mismatch at the boundary between the sample and the air.

4. Validity of the measurement of the dimensionless conductance *g*

In spite of a limited solid angle of the objective lens NA, we explain how the optical measurement of the dimensionless conductance g returns a reasonable value, based on the proposition that a localized mode is highly isotropic in angle[30-35]. Ideally, to completely measure g from a transmission matrix, the waves need to be incident onto the sample in all angular directions (i.e. hemispherical illumination) and the transmitted waves also need to be collected in all directions (i.e. hemispherical detection).

However, for localized samples, the solid angle for both input and output channels does not necessarily be 100 % to obtain a reasonable value of g, because the illumination from any angular directions can be coupled to localized modes (i.e. isotropic). We note that the light localization in silk is alternatively proven by (i) the scaling parameters (i.e. var(s_{ba}) and var(s_a)), (ii) the independency of the transmitted speckle pattern on the illumination positions (Fig. 4), and (iii) the small cavities inside a silk fibre at the gain-loss equilibrium (Fig. 5b and Fig. 6f).

To address the question whether the localized modes in silk is isotropic or not, it is important to directly analyse the waves in the localized modes. From the Fourier transformation of the electric field from the FEM computation (Fig. 3e), the isotropic scattering property of the localized modes in silk is clearly visible by the ring in **k**-space, in which the waves are scattered (or detected) with equal efficiency into all possible directions. Indeed, the cumulative behaviour of the medium depends strongly on the way that the scatterers are packed together; the scattering becomes more isotropic as the system becomes more disordered, regardless of the single scattering properties[30,34]. In this case, the illumination from any angular directions can be coupled to the localized modes. Because the current microscopy setup covers an angular passband (i.e. $\sin^{-1}$(NA)) of 64°, an additional increase in the illumination's solid angle at a fixed position would not result in excitation of an increased number of localized modes. Equivalently, an increase in the collection's solid angle under a given illumination condition would not detect an increased number of localized modes either. Given that g is an effective number of open transmission channels, this value would not change unless any additional speckle patterns are generated. A larger solid angle merely adds a constant prefactor in the speckle intensity[32,35], while keeping a value of $T_a(\alpha, \beta)$ (i.e. ratio of the transmitted to incident intensity) similar. Consequently, even accounting for the full solid angle in the localized specimen of silk, the value of g (i.e. cumulative sum of $T_a(\alpha, \beta)$) would be consistent.

5. Compensation of FTIR microscopy measurements

When thermal radiation strikes the silk surface, the reflected radiation is scattered in all directions. However, a FTIR microscope (Bruker HYPERION 1000) has a limited NA of 0.4 – 0.65 and only the angular passband (i.e. $\sin^{-1}(NA)$) of 23.6º – 40.5º was collected. To compensate for the loss by the limited NA in the FTIR microscope, we estimated a complete angular distribution of IR scattering using a customized gonio-ellipsometer system and the Henyey-Greenstein angular function[36]; the customized gonio-ellipsometer system was modified from an IR ellipsometer (J.A. Woollam IR-VASE). The FTIR spectrum captures the vibrational modes of silk protein. For visual clarity of these vibrational modes, the emissivity spectra are plotted as a function of both the wavelength (Fig. 7d) and the wavenumber (Supplementary Figure 12d). We note that the FTIR microscope provides a better spectral resolution in IR compared to the hemispherical measurement system (Surface optics SOC-100 HDR).

6. Measurements of the complex refractive index of regenerated silk

To characterize the fundamental dielectric response of silk protein with resonant absorptive processes, we measured the complex refractive index, of which the real part Re(n) describes the refraction and the imaginary part Im(n) describes the extinction. For refractive index measurements, we synthesized a uniform and transparent regenerated silk film with a thickness of 90 μm (inset in Fig. 7e)[8,37]. In the synthesis, the processes of thermal annealing and UV exposure were excluded to minimize changes in the secondary structures (e.g. β-sheet, random coil, and α-helix) that might shift the exact peak positions of absorption. At all of the wavelengths in the entire visible/NIR/IR regions (λ = 0.4 – 20 μm), we obtained the complex refractive indices of the regenerated silk film using two different ellipsometers (J.A. Woollam V-VASE and IR-VASE). In the visible and NIR region (λ = 0.4 – 2 μm), Re(n) can be empirically expressed by Sellmeier equation:

$$\mathrm{Re}(n(\lambda))^2 - 1 = \frac{0.03329\lambda^2}{\lambda^2 - 0.1015} + \frac{1.277\lambda^2}{\lambda^2 - 0.01136}, \tag{7}$$

and Im(n) is close to zero (left panel of Fig. 7e). In the IR region (λ = 2 – 20 μm), Re(n) + iIm(n) as a function of photon energy E (= 1.24/λ) can be modelled with multiple Lorentz oscillators:

$$\begin{aligned}(\mathrm{Re}(n(E)) + i\,\mathrm{Im}(n(E)))^2 &= \frac{A_1}{E_1^2 - E^2 - i\gamma_1 E} + \frac{A_2}{E_2^2 - E^2 - i\gamma_2 E} + \frac{A_3}{E_3^2 - E^2 - i\gamma_3 E} + \frac{A_4}{E_4^2 - E^2 - i\gamma_4 E} \\ &+ \frac{A_5}{E_5^2 - E^2 - i\gamma_5 E} + \frac{A_6}{E_6^2 - E^2 - i\gamma_6 E} + \frac{A_7}{E_7^2 - E^2 - i\gamma_7 E} + \frac{A_8}{E_8^2 - E^2 - i\gamma_8 E} + \frac{A_9}{E_9^2 - E^2 - i\gamma_9 E} \\ &+ \frac{A_{10}}{E_{10}^2 - E^2 - i\gamma_{10} E} + \frac{A_{11}}{E_{11}^2 - E^2 - i\gamma_{11} E} + \frac{A_{12}}{E_{12}^2 - E^2 - i\gamma_{12} E} + \frac{A_{13}}{E_{13}^2 - E^2 - i\gamma_{13} E},\end{aligned} \tag{8}$$

where A_h, E_h, and γ_h are the amplitude, the central energy, and the linewidth of hth Lorentz oscillator, respectively. In this oscillator model, Re(n) and Im(n) are connected through the Kramers-Kronig relation and this classical representation is adequate for describing resonant absorptive processes. The results for fitted model parameters, which were used to plot right panel of Fig. 7e, are summarized in the following table:

h	Amplitude A_h	Central energy E_h [eV]	Linewidth γ_h [eV]
1	0.40408 ± 0.214	0.063315 ± 0.0135	0.017748 ± 0.0455
2	0.17157 ± 0.465	0.07 ± 0.00452	0.005878 ± 0.0178
3	0.4586 ± 0.319	0.083493 ± 0.00265	0.022808 ± 0.00877
4	0.2 ± 0.115	0.13049 ± 0.00184	0.0090362 ± 0.00339
5	0.36671 ± 0.196	0.15513 ± 0.000604	0.01 ± 0.00206
6	-0.017132 ± 0.0936	0.35049 ± 0.0342	0.0020067 ± 0.0144
7	0.22238 ± 0.123	0.17203 ± 0.000998	0.01 ± 0.00327
8	0.90845 ± 0.481	0.19051 ± 0.000182	0.0066803 ± 0.00056
9	1.5017 ± 0.79	0.20481 ± 9.98e-005	0.0061377 ± 0.000214
10	0.067788 ± 0.0408	0.36412 ± 0.0116	0.04091 ± 0.0289
11	0.07 ± 0.0804	0.38256 ± 0.00378	0.0052526 ± 0.00681
12	0.42159 ± 0.221	0.40935 ± 0.000701	0.021749 ± 0.00216
13	0.044237 ± 0.0229	0.66417 ± 0.0315	0.30463 ± 0.102

Supplementary Figures

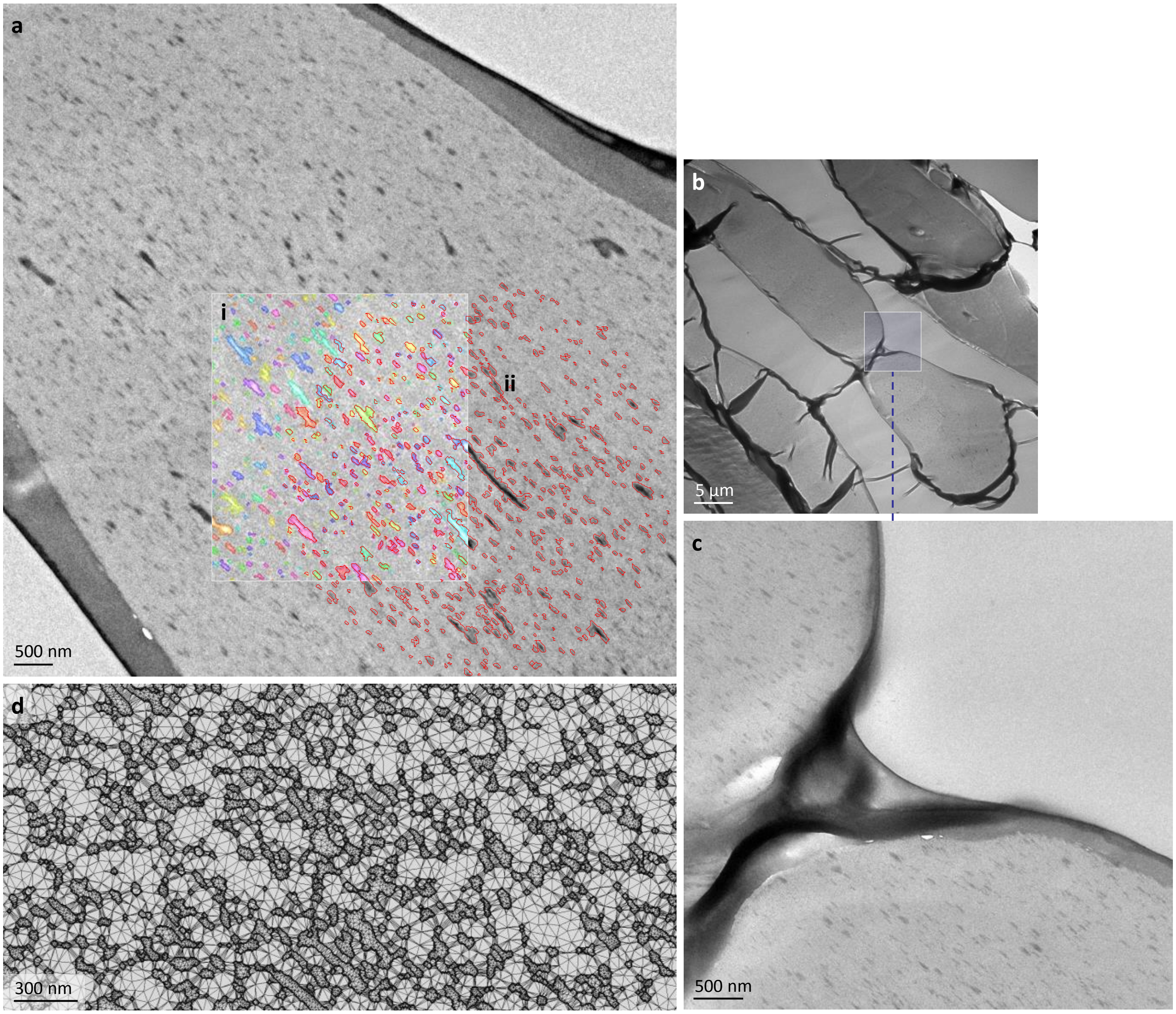

Supplementary Figure 1. Post-processing of TEM images to extract nanofibrillar boundaries and domains for FEM computations.

a, Extraction of the boundaries and domains of nanofibrils, interfibrillar areas, and sericin from TEM micrographs for FEM computations. The dark granule-like dots indicate nanofibrils resulting from higher electron density of metal-stained nanofibrils than that of voids. The non-uniform background intensity in the filament is estimated using a morphological opening algorithm and is subtracted from the raw image. Then, a binary image is generated by increasing its image contrast and applying a

threshold. From the binary image, connected components are identified, marked as the different pseudo-colours in (i). Using the connected components, the region boundaries are traced with the red lines in (ii). The volume fraction of nanofibrils is ~ 30%, which is defined as a fraction of the area occupied by the nanofibrils to the total area of the binary image. **b**, TEM micrograph of the transverse cross-section of two fibroin filaments in the silk fibre. **c,** Higher magnification TEM micrograph from the area marked by the rectangular box in **b** shows the presence of nanofibrils (dark granule-like dots) inside the fibroin filament and the absence outside the sericin layer. **d,** Representative boundaries and triangular meshes used in FEM computations. The thick black lines represent the boundaries of nanofibrils. The maximal mesh sizes are set to $\lambda/10$ in the interfibrillar areas and are reduced to $\lambda/20$ inside the nanofibrils, due to their complex shapes.

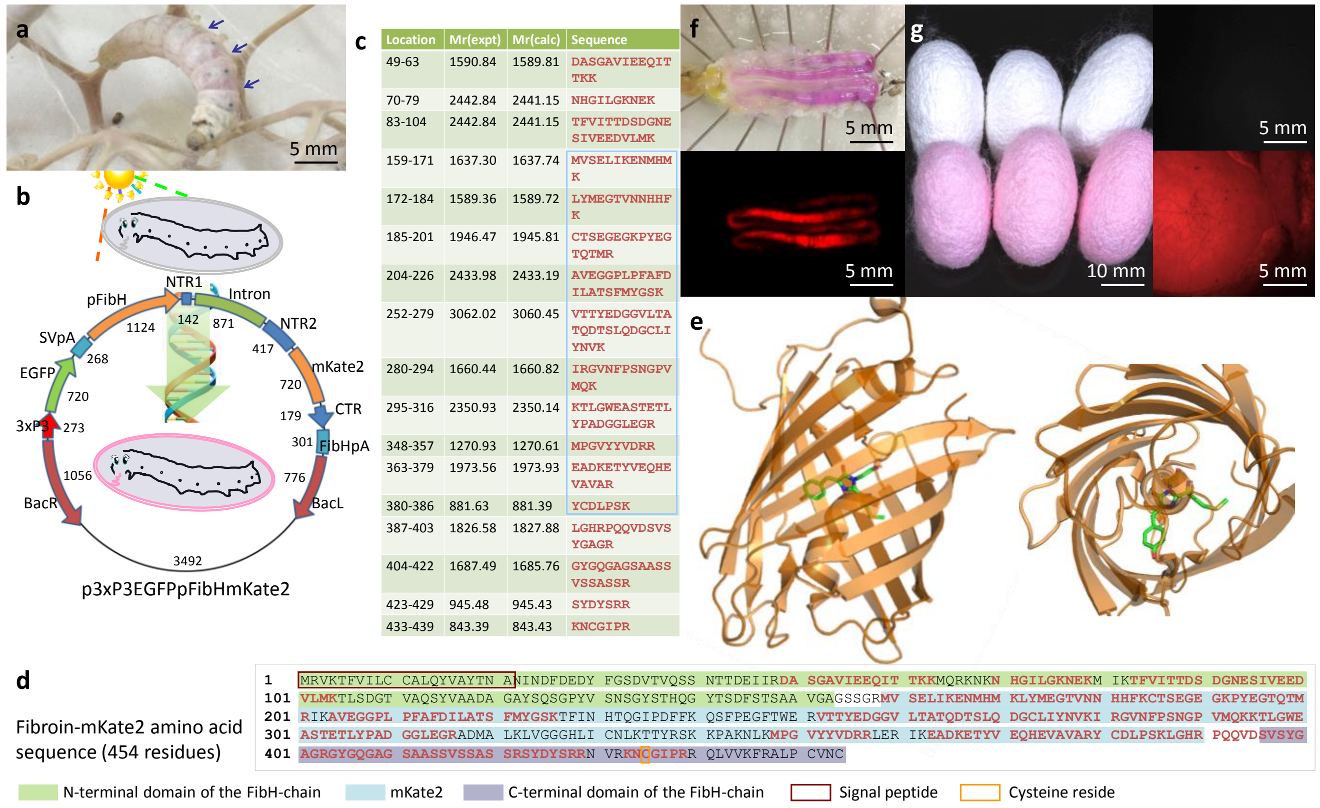

Supplementary Figure 2. Silkworm transgenesis for molecular imaging of nanofibrils.

a, Photograph of a transgenic silkworm in the rearing environment. The colour of FibH chain-mKate2 fusion proteins (arrows) appears beneath the epidermis. **b**, Physical map of the transformation vector of p3xP3-EGFP-pFibH-mKate2. **c**, Identification of FibH chain-mKate2 fusion proteins by high-performance liquid chromatography with tandem mass spectrometry (LC-MS/MS). *De novo* sequences of tryptic fragments Mr(expt) are confirmed to match with the deduced amino acid sequences of FibH chain-mKate2 fusion proteins Mr(calc). **d**, Amino acid sequences of FibH chain-mKate2 fusion proteins. **e**, Stereoview of the chromophore (green sticks) in the crystal structure of mKate2 at a physiological pH of 7.5, visualized by using PyMOL (PDB: 3SVR). mKate2 is a S158A variant of mKate that replaces Ser^{158} of mKate by a hydrophobic residue, causing partial destabilization of the dimmer *trans* state of chromophore. This induces the equilibrium toward the brighter *cis* state (green sticks), thus being more pH-stable and increasing the brightness at a physiological pH. **f**, Photograph (upper) and fluorescent image (lower) of the silk gland of the larva

on the 3rd day of the 5th instar. **g**, Photograph of the white (upper) and fluorescent (lower) silk cocoons produced by wild-type and mKate2-expressing silkworms and corresponding fluorescent images.

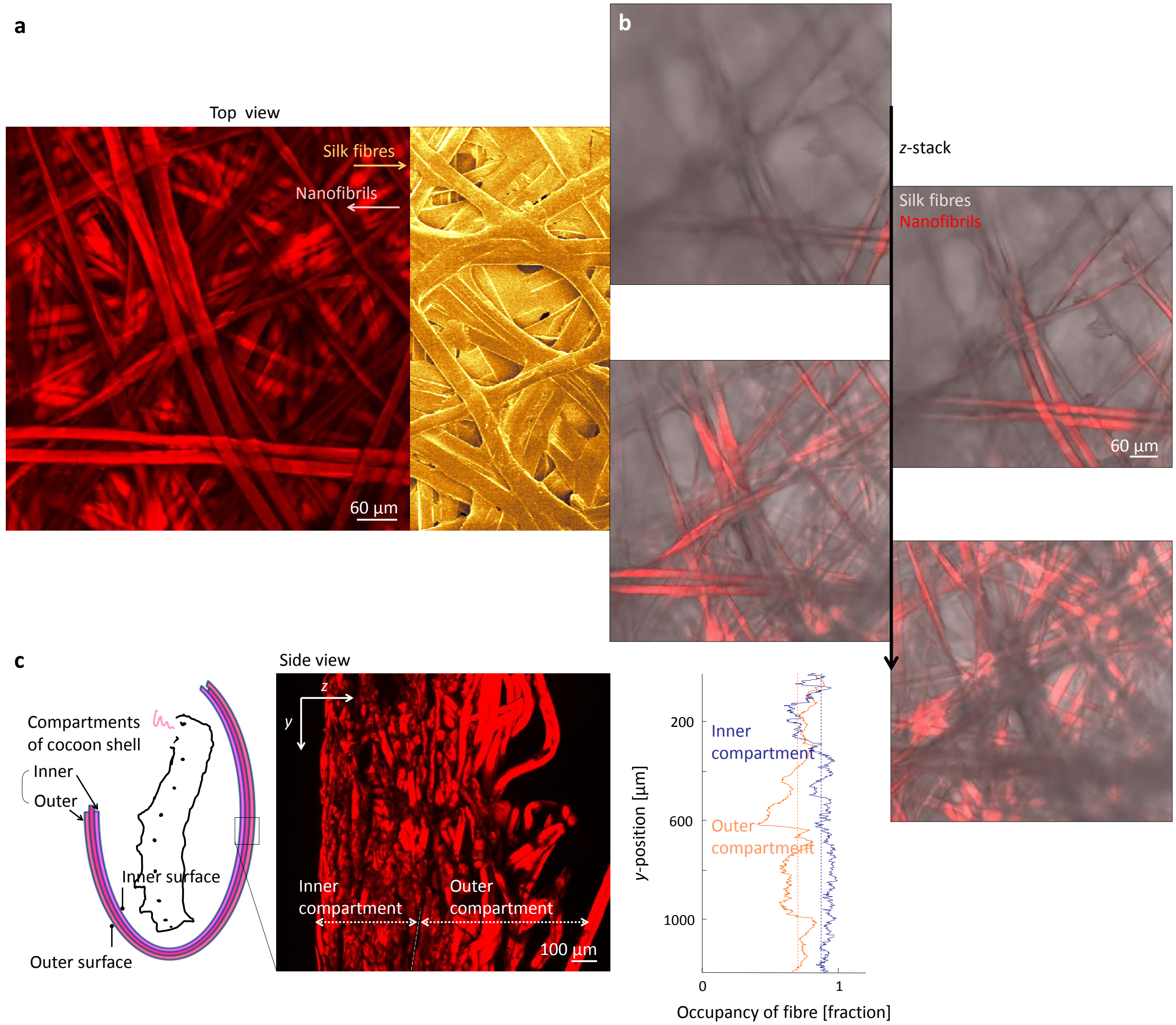

Supplementary Figure 3. Molecular imaging of nanofibrils, showing the distribution of nanofibrils on microscales.

a, Left: Confocal fluorescence microscopy image of mKate2-expressing silk nanofibrils. Right: SEM image of the silk fibre surfaces. **b**, Serial optical sections with a step size of 14 µm. Reflectance (gray) and fluorescence (red) microscopy images are overlaid. The silk fibres are composed of nanofibrils, forming the highly packed multilayered microstructures of silk cocoons. **c**, Cross-section of the silk cocoon. Left: Compartments of the cocoon shell. Middle: Confocal fluorescence microscopy image of the cross-section. Right: Occupancy of silk fibres over *z*-axis. The silk fibres of the inner

compartment are more uniformly and highly packed than those of the outer compartment. Thus, the inner compartment of native silk cocoons is used for the optical measurements (Fig. 4 and 5).

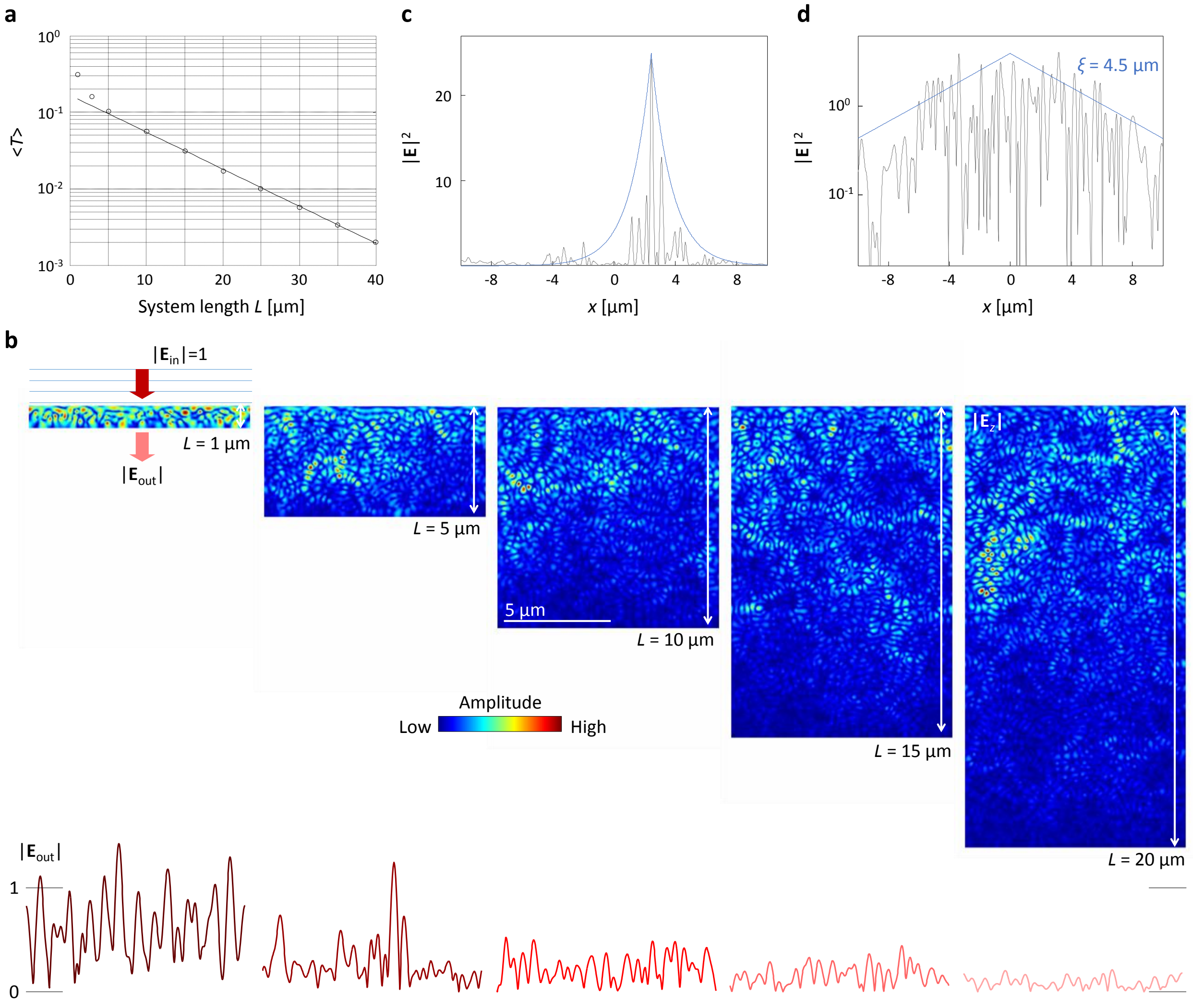

Supplementary Figure 4. Calculations of the localization length of light ξ.

a, Computed average transmittance $< T >$ through the nanofibrillar system (representative domains are shown in Fig. 1d and Supplementary Figure 1a). For a large system length of $L > 5$ µm, the decay over L converges to a single exponential and fitting a slope of $< \ln T > \approx -L/2\xi$ carries out $\xi = 4.5$ µm. **b**, Computations of plane wave propagation using FEM, through the nanofibrillar system of L = 1, 5, 10, 15, and 20 µm. The plane wave at λ = 600 nm with unit amplitude is launched at the top plane. For visual clarity, the amplitude is displayed rather than the intensity. The transmitted amplitude patterns $|E_{out}|$ at the output plane are shown by the red curves (bottom). **c**, **d**, Field intensity profiles along the

white lines marked by i (**c**) and ii (**d**) in Fig. 2a. The blue curves are obtained by Supplementary Eq. (3) with the estimation of ξ from an inverse participation ratio (Supplementary Eq. (2)).

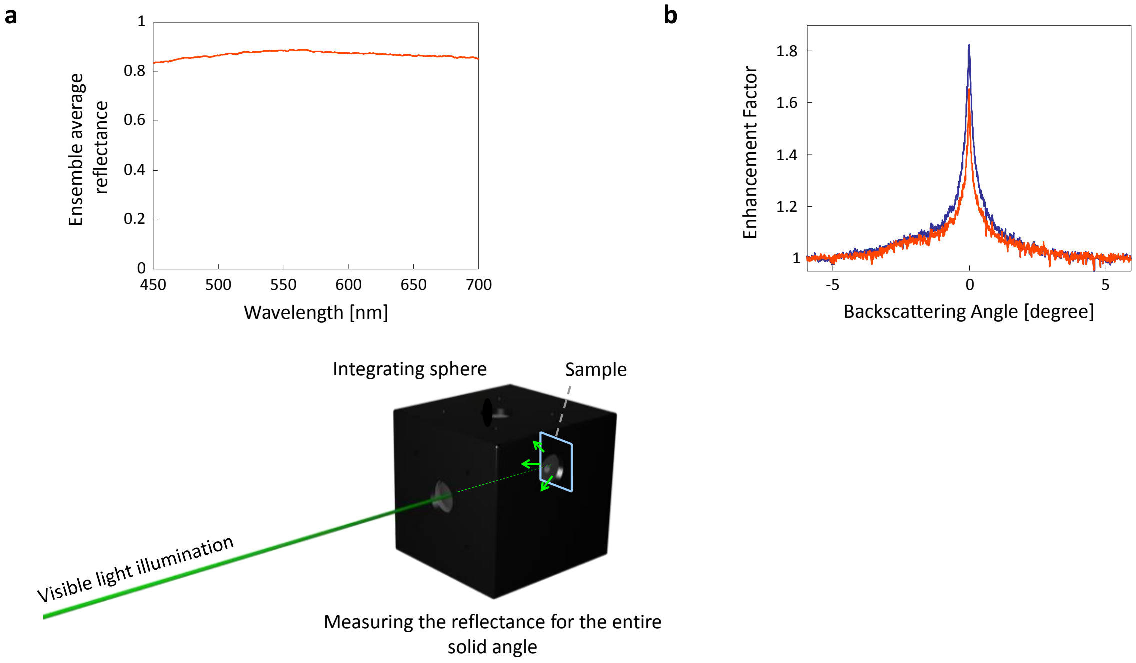

Supplementary Figure 5. Measurements of the scattering properties.

a, Top: Ensemble averaged reflectance from an entire white silk cocoon, calibrated with a white reflectance standard with a reflectivity of 99% (Labsphere SRS-99-010). Bottom: Schematics of reflectance measurements for the entire solid angle Ω using an integrating sphere (Thorlabs IS200-4). **b**, Measurements of the transport mean free path length l_t from coherent backscattering of light. A beam with a diameter of 3 mm from a He-Ne laser at 543.5 nm is illuminated onto the specimen on a rotating stage. The angular profiles of coherent backscattering return $l_t = 16 \pm 0.2$ µm and 7.3 ± 0.1 µm for the white silk cocoon (red curve) and white paper (blue curve) specimens, respectively.

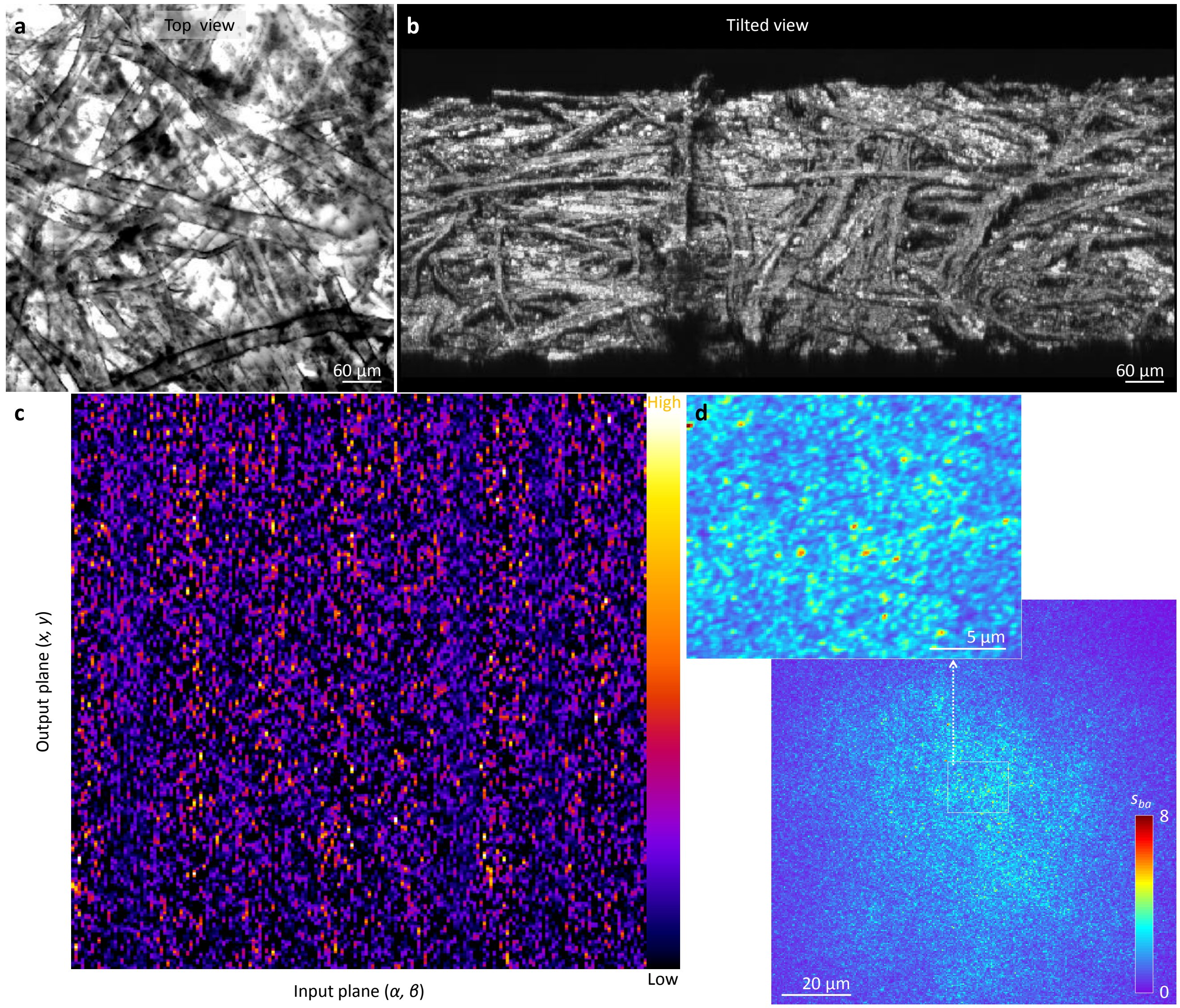

Supplementary Figure 6. Disappearance of light localization when fibres lose nanofibrils.

a, b, Reflectance confocal microscopy images of the white paper specimen in top (**a**) and tilted (**b**) views. **c,** TM of the white paper specimen after removing the fluctuation components originated from the background local sample inhomogeneity. Each column of TM is a rearranged matrix of $T_{ba}(x, y)$ recorded at a position of (α, β). **d,** Representative $s_{ba}(x, y)$ maps. The uniformity of speckle patterns is captured as a Rayleigh distribution in $P(s_{ba})$ in the inset of Fig. 4c. The waves are diffusive with an estimated value of $g = 128.5$, as predicted in Fig. 2b.

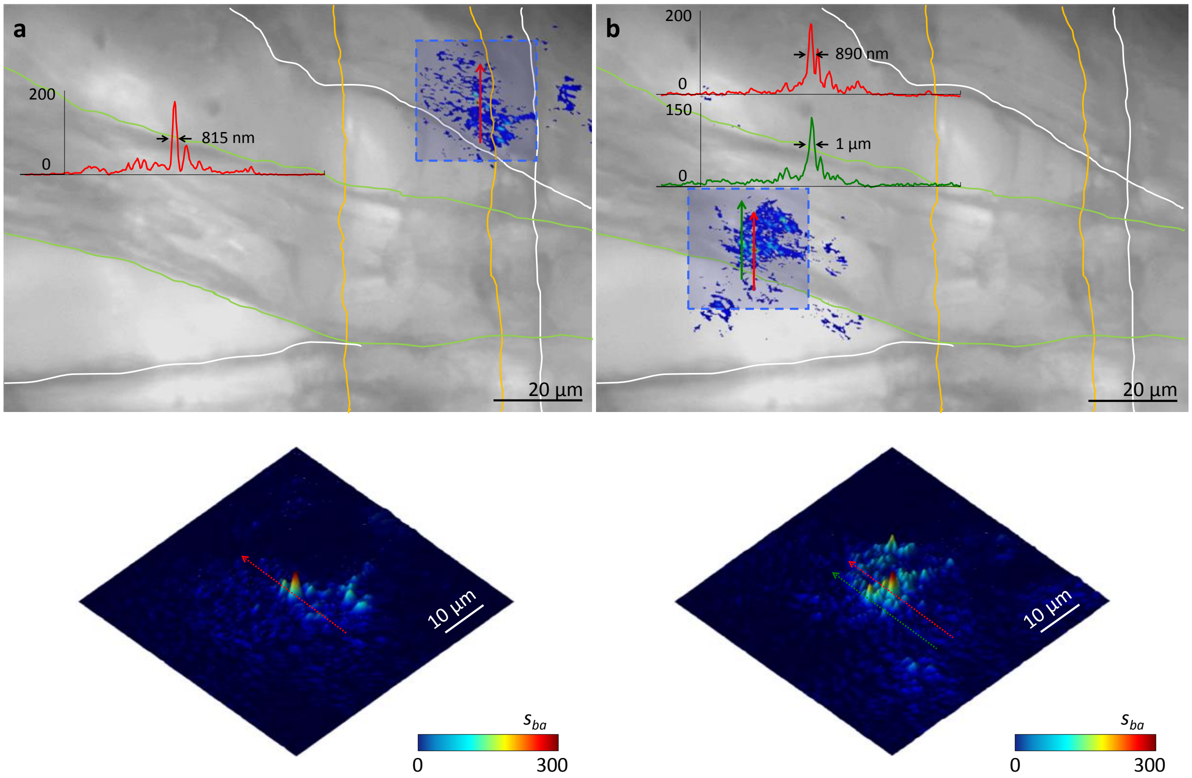

Supplementary Figure 7. Extra tunnelling locations in Fig. 4f.

a, **b**, Resonance tunnelling occurs on the stacks of filaments marked by white (**a**) and green (**b**) boundaries. The transmission is near zero for other locations of the input illumination in the field of view of Fig. 4f. The background (grayscale image) is a microscopy image of the surface of the silk cocoon obtained by incoherent white-light large-area illumination. The speckle images ($s_{ba}(x, y)$ maps in pseudo-colour) and the grayscale image are taken on the same focal plane of the most outer surface of the silk fibres. The surface plots of $s_{ba}(x, y)$ correspond to the areas marked by the blue-shaded boxes with the cross-sections at their highest peaks (along the coloured arrows on the surface plots) for visual clarity.

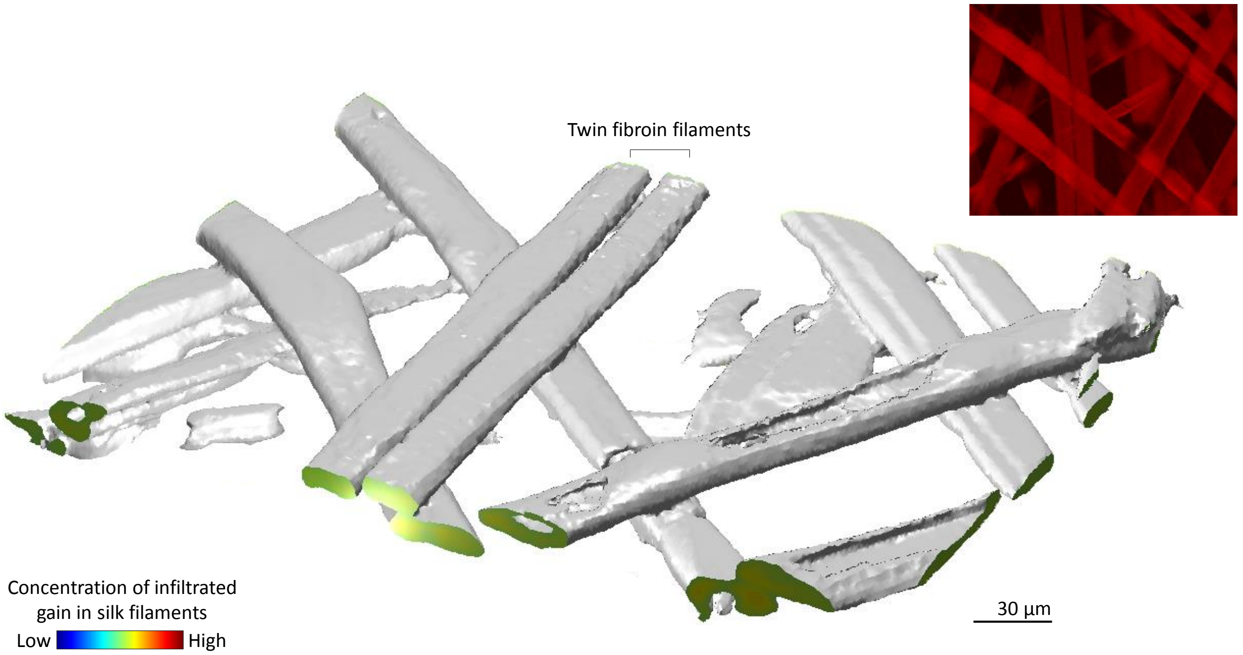

Supplementary Figure 8. Internal luminescence embedded in silk fibres.

3D rendering of silk fibroin filaments infiltrated with DCM. The isosurface (gray colour) represents a level set of gain concentrations in the reconstructed volume. The cross-sections (pseudo-colour map) show the homogenous distribution of gain, enabling for compensating the system loss and characterizing the modes inside the filaments. The reconstructed 3D concentration of gain is obtained by a *z*-stack of confocal fluorescence microscopy images (top right: 2D projection of images over the *z*-axis).

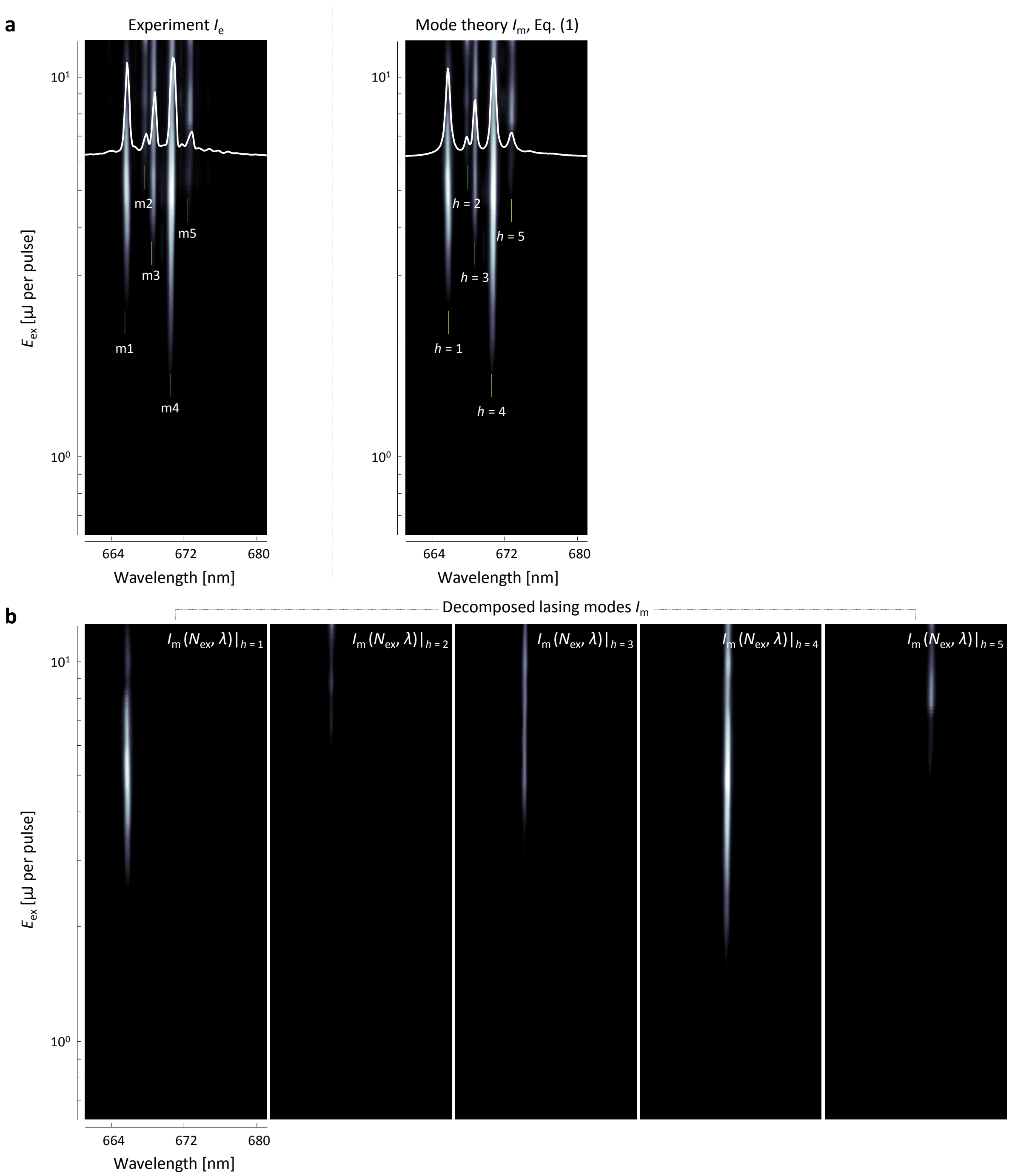

Supplementary Figure 9. Modal decompositions of $I_e(E_{ex}, \lambda)$.

a, Left: Measured $I_e(E_{ex}, \lambda)$ from the silk cocoon. The spectra show five non-interacting lasing peaks marked with m1 – m5. Right: Spectra $I_m(E_{ex}, \lambda)$ obtained by substituting the inverse estimations of a_h, ω_h, and Γ_h to the analytical expression of Eq. (1) with a total number of modes of $H = 5$. The cross-sections at $E_{ex} = 6.2$ μJ/pulse (white lines) are plotted for comparison between the fitted spectra and the

measured spectra. **b**, Spectral evolution of decomposed single lasing modes as a function of E_{ex}. Five lasing modes have their consistent central wavelengths λ_h, while each shows threshold behaviour.

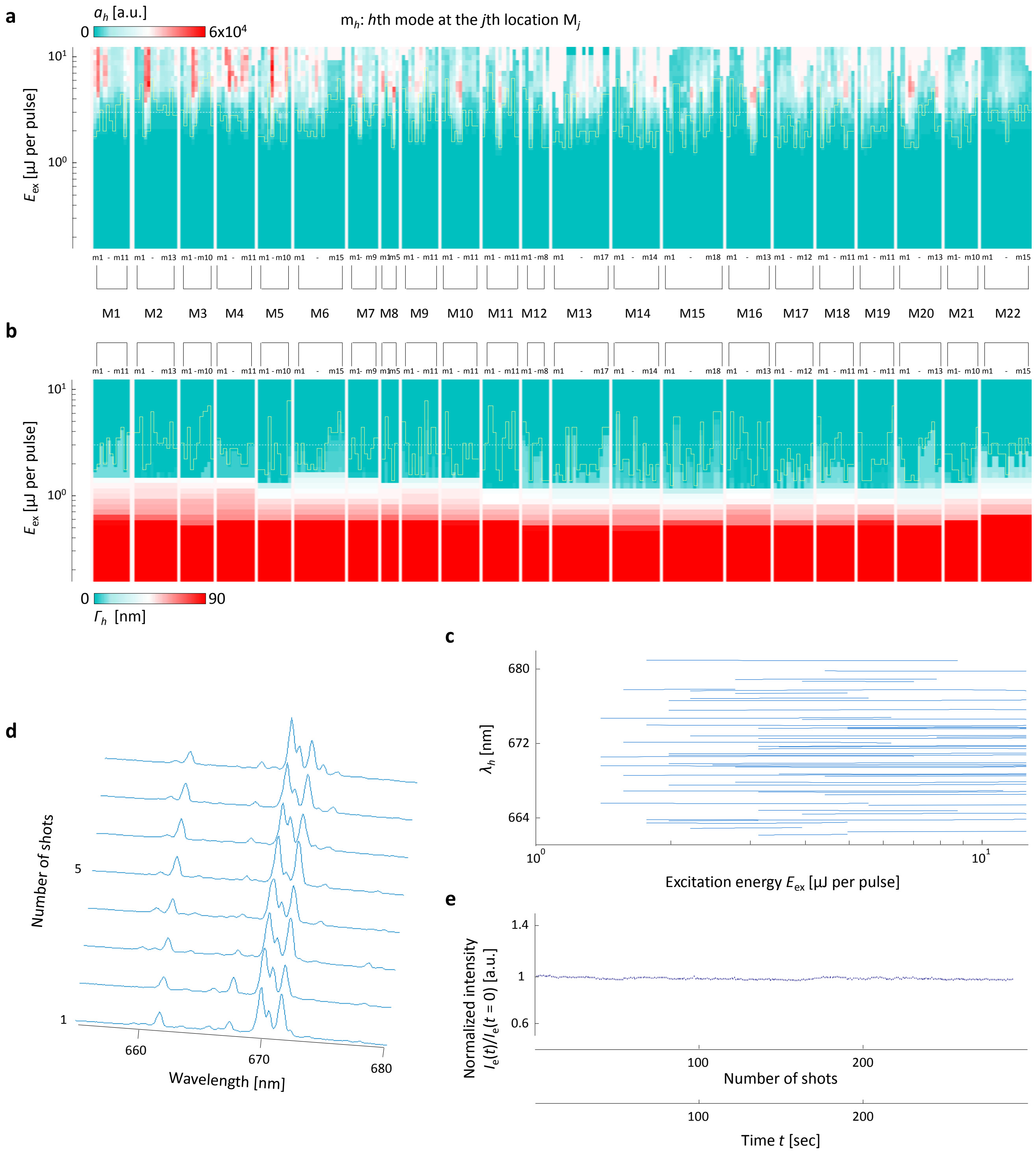

Supplementary Figure 10. *a_h*, *Γ_h*, and *λ_h* from modal decompositions.

a–**c**, Intensity a_h (**a**), linewidth Γ_h in λ (**b**), and central wavelength λ_h (**c**) of lasing modes decomposed from $I_e(E_{ex}, \lambda)$. A total of 259 modes acquired at 22 different locations with a translation step of > 100 μm on the silk cocoon are analyzed. Each column of **a** and **b** indicates the evolution of a_h and Γ_h for

the m_h single lasing mode as a function of E_{ex}. The Q factors ($= \omega_h/\Gamma_h$) are evaluated at the threshold of each decomposed mode (yellow solid lines). The white dashed lines mark the average lasing thresholds of the silk specimen (Fig. 6c). The data in **a**−**c** are used for the analyses in Fig. 6a−e. **d**, Spectra from single-shot excitation pulses at E_{ex} = 3.5 μJ/pulse in a fixed location. The series of single-shot spectra are almost identical. **e**, Shot-to-shot fluctuations of emission intensity. The emission intensity, normalized by the initial emission intensity $I_e(t)/I_e(t = 0)$, is stable over 300 seconds (or 300 successive excitation pulses).

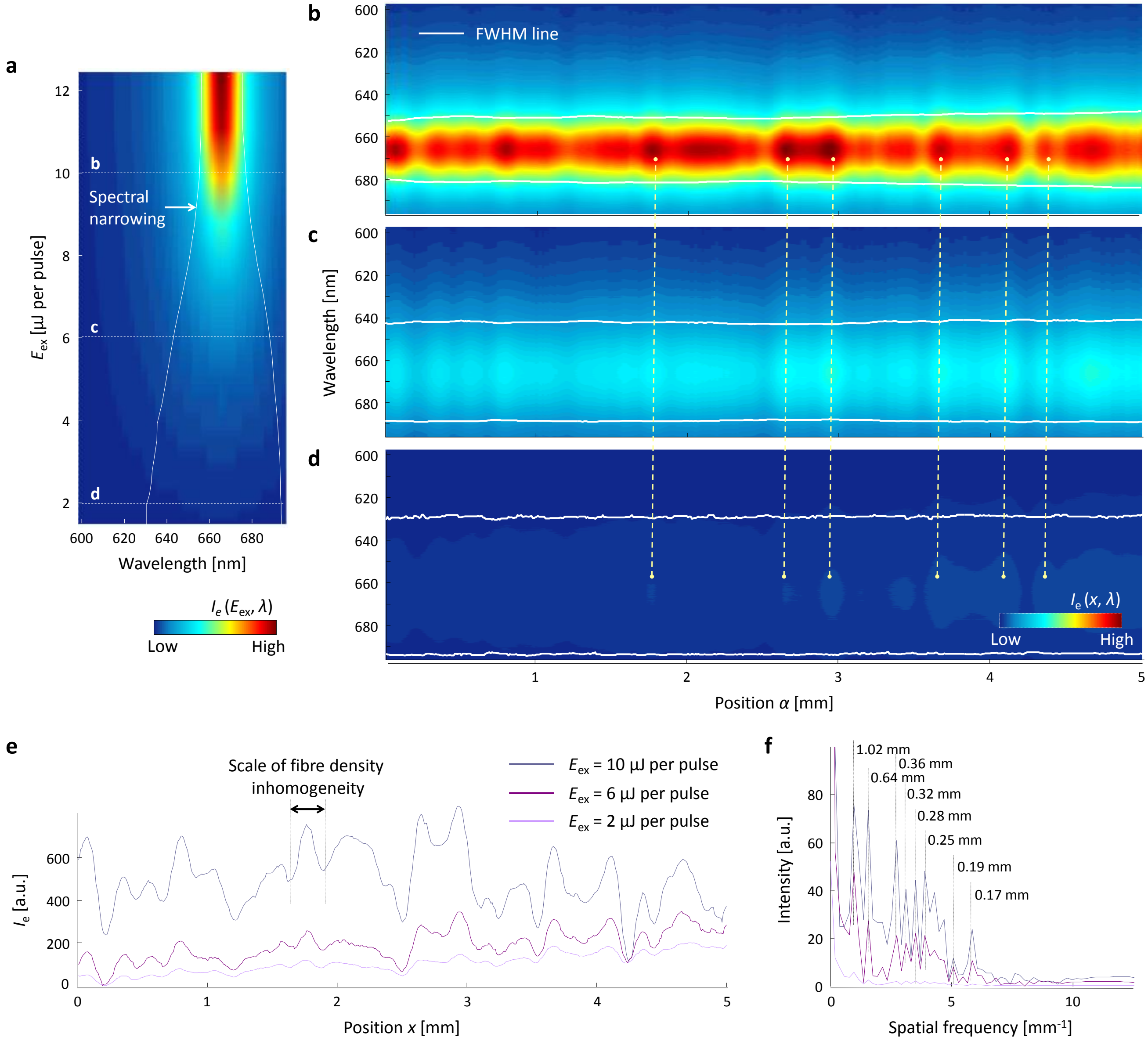

Supplementary Figure 11. Disappearance of localized modes when fibres lose nanofibrils.

a, $I_e(E_{ex}, \lambda)$ from the DCM-infiltrated paper specimen. The white solid lines mark the FWHMs of spectra that capture spectral narrowing of diffusive lasing (or amplified spontaneous emission). **b−d,** $I_e(\mathbf{r}, \lambda)$ at three different levels of excitation energy E_{ex} = 10, 6, and 2 μJ/pulse, marked in **a**. The FWHMs (white solid lines) are similar in spatial positions, while the emission intensity fluctuates. **e,** Spatial emission patterns in **b−d** at λ = 665 nm. The positions of peaks and lobes are nearly consistent over E_{ex} and the overall emission intensity increases simultaneously, because the emission fluctuation is originated from

the local sample inhomogeneity. Analysing distances between the lobes, the average length scale of fibre density inhomogeneity in the paper is ~ 215 µm. **f**, Spatial Fourier transforms of **e**. The spatial frequencies capture various length scales of fluctuations ranging from 170 µm to 1 mm.

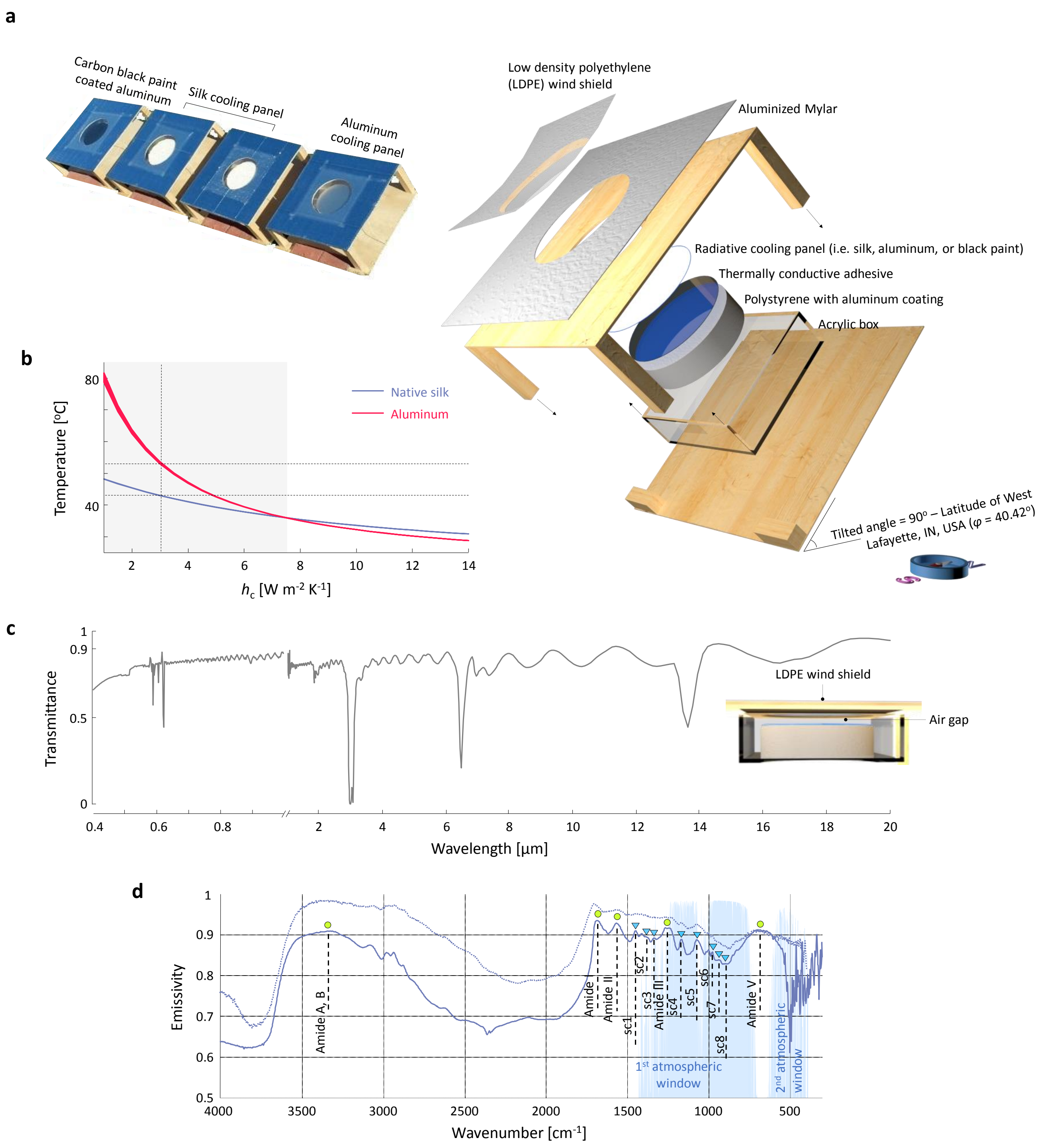

Supplementary Figure 12. Biophotonic cooling device.

a, Photograph of the biophotonic cooling device (i.e. native silk) and reference testbeds, which are composed of conventional reflecting (e.g. aluminum) and high-emissive (e.g. carbon black paint-coated aluminum) materials. To selectively characterize passive radiative cooling in native silk, the

experimental configuration is designed to minimize the measurement uncertainty by reducing the thermodynamic complexity (Methods). **b**, Computed steady-state temperature of the silk and aluminum panels at the peak sunlight (2 PM local time) as a function of the convective heat transfer coefficient h_c. Linear regression with the measured data (dashed horizontal lines) returns h_c = 3 W m^{-2} K^{-1} such that the low-density polyethylene (LDPE) film indeed suppress air flow around the cooling panel. Thus, the effect of passive radiative cooling becomes prominent to be measurable. **c**, Transmittance of the LDPE film with a thickness of 19 μm, which is placed on top surface of the testbed to seal air flow around the cooling panel. **d**, Ensemble averaged emissivity of native silk as a function of the wavenumber measured with the FTIR microscope (blue solid line) and the hemispherical directional reflectometer (HDR) (blue dotted line) systems. The information on the marks and the labels are summarized in Fig. 7d.

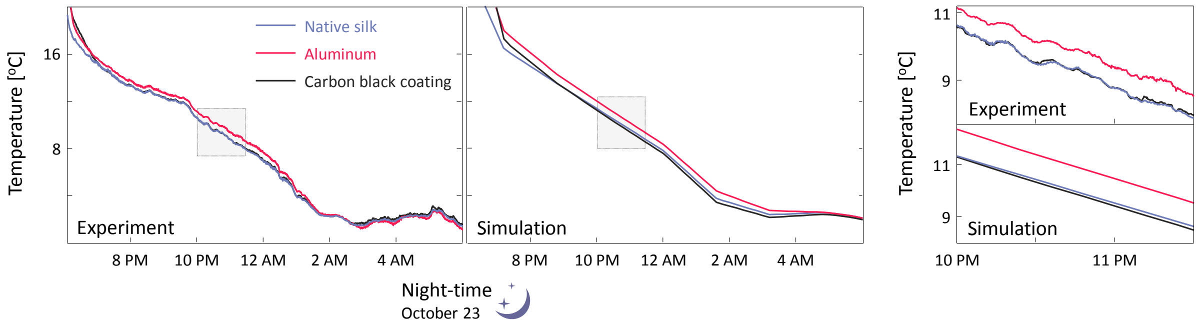

Supplementary Figure 13. Night-time radiative cooling.

Measured and computed steady-state temperature of native silk, aluminum, and carbon black paint-coated aluminum after the Sun sets beyond the horizon (6 PM local time). These extend the data from Fig. 7a. The gain terms of the radiative heat source in the 3D heat equation becomes negligible by the disappearance of sunlight. Thus, the cooling effect at night under a clear sky is purely determined by the emissivity in IR (Eq. (4)). This prediction is in good agreement with the outdoor measurements at night under a clear sky. We note that the emissivity of the carbon black paint (Krylon 1602 ultra flat black) is close to unity in IR.

Supplementary References

1 Drummy, L. F., Phillips, D. M., Koerner, H., Vaia, R. A., Farmer, B. L. & Naik, R. R. Low voltage electron microscopy of silk fibers and films. *Microscopy and Microanalysis* 11, 1268-1269, (2005).

2 Hakimi, O., Knight, D. P., Knight, M. M., Grahn, M. F. & Vadgama, P. Ultrastructure of insect and spider cocoon silks. *Biomacromolecules* 7, 2901-2908, (2006).

3 Trancik, J. E., Czernuszka, J. T., Bell, F. I. & Viney, C. Nanostructural features of a spider dragline silk as revealed by electron and X-ray diffraction studies. *Polymer* 47, 5633-5642, (2006).

4 Shcherbo, D., Murphy, C. S., Ermakova, G. V., Solovieva, E. A., Chepurnykh, T. V., Shcheglov, A. S., Verkhusha, V. V., Pletnev, V. Z., Hazelwood, K. L., Roche, P. M., Lukyanov, S., Zaraisky, A. G., Davidson, M. W. & Chudakov, D. M. Far-red fluorescent tags for protein imaging in living tissues. *Biochemical Journal* 418, 567-574, (2009).

5 Tamura, T., Thibert, C., Royer, C., Kanda, T., Abraham, E., Kamba, M., Komoto, N., Thomas, J. L., Mauchamp, B., Chavancy, G., Shirk, P., Fraser, M., Prudhomme, J. C. & Couble, P. Germline transformation of the silkworm *Bombyx mori* L. Using a *piggyBac* transposon-derived vector. *Nature Biotechnology* 18, 81-84, (2000).

6 Teule, F., Miao, Y. G., Sohn, B. H., Kim, Y. S., Hull, J. J., Fraser, M. J., Lewis, R. V. & Jarvis, D. L. Silkworms transformed with chimeric silkworm/spider silk genes spin composite silk fibers with improved mechanical properties. *Proceedings of the National Academy of Sciences of the United States of America* 109, 923-928, (2012).

7 Iizuka, T., Sezutsu, H., Tatematsu, K., Kobayashi, I., Yonemura, N., Uchino, K., Nakajima, K., Kojima, K., Takabayashi, C., Machii, H., Yamada, K., Kurihara, H., Asakura, T., Nakazawa, Y., Miyawaki, A., Karasawa, S., Kobayashi, H., Yamaguchi, J., Kuwabara, N., Nakamura, T.,

Yoshii, K. & Tamura, T. Colored fluorescent silk made by transgenic silkworms. *Advanced Functional Materials* 23, 5232-5239, (2013).

8 Kim, D. W., Lee, O. J., Kim, S. W., Ki, C. S., Chao, J. R., Yoo, H., Yoon, S. I., Lee, J. E., Park, Y. R., Kweon, H., Lee, K. G., Kaplan, D. L. & Park, C. H. Novel fabrication of fluorescent silk utilized in biotechnological and medical applications. *Biomaterials* 70, 48-56, (2015).

9 Kim, S. W., Yun, E. Y., Choi, K. H., Kim, S. R., Kang, S. W., Park, S. W. & Goo, T. W. Utilization of the *Bombyx mori* heat shock protein 70 promoter for screening transgenic silkworms. *Entomological Research* 43, 282-287, (2013).

10 Milner, V. & Genack, A. Z. Photon localization laser: Low-threshold lasing in a random amplifying layered medium via wave localization. *Physical Review Letters* 94, 073901, (2005).

11 Pena, A., Girschik, A., Libisch, F., Rotter, S. & Chabanov, A. A. The single-channel regime of transport through random media. *Nature Communications* 5, 3488, (2014).

12 Choi, S. H., Byun, K. M. & Kim, Y. L. Excitation of multiple resonances in 1D Anderson localized systems for efficient light amplification. *Optics Letters* 40, 847-850, (2015).

13 Schwartz, T., Bartal, G., Fishman, S. & Segev, M. Transport and Anderson localization in disordered two-dimensional photonic lattices. *Nature* 446, 52-55, (2007).

14 Stano, P. & Jacquod, P. Suppression of interactions in multimode random lasers in the Anderson localized regime. *Nature Photonics* 7, 66-71, (2013).

15 Busch, K., Soukoulis, C. M. & Economou, E. N. Transport and scattering mean free paths of classical waves. *Physical Review B* 50, 93-98, (1994).

16 van Rossum, M. C. W. & Nieuwenhuizen, T. M. Multiple scattering of classical waves: Microscopy, mesoscopy, and diffusion. *Reviews of Modern Physics* 71, 313-371, (1999).

17 Lenke, R. & Maret, G. in *Scattering in polymeric and colloidal systems* (eds W. Brown & K. Mortensen) (CRC Press 2000).

18 Conley, G. M., Burresi, M., Pratesi, F., Vynck, K. & Wiersma, D. S. Light transport and localization in two-dimensional correlated disorder. *Physical Review Letters* 112, 143901, (2014).

19 van Albada, M. P. & Lagendijk, A. Observation of weak localization of light in a random medium. *Physical Review Letters* 55, 2692-2695, (1985).

20 Wolf, P. E. & Maret, G. Weak localization and coherent backscattering of photons in disordered media. *Physical Review Letters* 55, 2696-2699, (1985).

21 Wiersma, D. S., Bartolini, P., Lagendijk, A. & Righini, R. Localization of light in a disordered medium. *Nature* 390, 671-673, (1997).

22 Liu, J., Xu, Z., Song, Q., Konger, R. L. & Kim, Y. L. Enhancement factor in low-coherence enhanced backscattering and its applications for characterizing experimental skin carcinogenesis. *Journal of Biomedical Optics* 15, 037011, (2010).

23 Tuchin, V. V. *Tissue optics: Light scattering methods and instruments for medical diagnosis*. 3 edn, (SPIE-The International Society for Optical Engineering, 2015).

24 Ntziachristos, V. Going deeper than microscopy: The optical imaging frontier in biology. *Nature Methods* 7, 603-614, (2010).

25 Onufrak, M. A. V., Konger, R. L. & Kim, Y. L. Telecentric suppression of diffuse light in imaging of highly anisotropic scattering media. *Optics Letters* 41, 143-146, (2016).

26 Prahl, S. A., van Gemert, M. J. C. & Welch, A. J. Determining the optical properties of turbid media by using the adding doubling method. *Applied Optics* 32, 559-568, (1993).

27 Zhu, J. X., Pine, D. J. & Weitz, D. A. Internal reflection of diffusive light in random-media. *Physical Review A* 44, 3948-3959, (1991).

28 Lagendijk, A., Vreeker, R. & Devries, P. Influence of internal reflection on diffusive transport in strongly scattering media. *Physics Letters A* 136, 81-88, (1989).

29 Denouter, P. N. & Lagendijk, A. Influence of the refractive-index contrast on coherent backscattering. *Optics Communications* 103, 169-173, (1993).

30 Wiersma, D. S., Sapienza, R., Mujumdar, S., Colocci, M., Ghulinyan, M. & Pavesi, L. Optics of nanostructured dielectrics. *Journal of Optics A: Pure and Applied Optics* 7, S190-S197, (2005).

31 Andreasen, J., Asatryan, A. A., Botten, L. C., Byrne, M. A., Cao, H., Ge, L., Labonté, L., Sebbah, P., Stone, A. D., Türeci, H. E. & Vanneste, C. Modes of random lasers. *Advances in Optics and Photonics* 3, 88-127, (2011).

32 Shi, Z. & Genack, A. Z. Transmission eigenvalues and the bare conductance in the crossover to Anderson localization. *Physical Review Letters* 108, 043901, (2012).

33 Choi, W., Park, Q. H. & Choi, W. Perfect transmission through Anderson localized systems mediated by a cluster of localized modes. *Optics Express* 20, 20721-20729, (2012).

34 Cao, H. & Noh, H. in *Amorphous Nanophotonics* (eds C. Rockstuhl & T. Scharf) (Springer-Verlag Berlin Heidelberg, 2013).

35 Leseur, O., Pierrat, R., Saenz, J. J. & Carminati, R. Probing two-dimensional Anderson localization without statistics. *Physical Review A* 90, 053827, (2014).

36 Henyey, L. G. & Greenstein, J. L. Diffuse radiation in the galaxy. *Astrophysical Journal* 93, 70-83, (1941).

37 Rockwood, D. N., Preda, R. C., Yucel, T., Wang, X. Q., Lovett, M. L. & Kaplan, D. L. Materials fabrication from *Bombyx mori* silk fibroin. *Nature Protocols* 6, 1612-1631, (2011).
