# Supplementary material for: Anderson light localization in biological nanostructures of native silk
Source: Nat Commun. 2018 Jan 31;9:452. doi: 10.1038/s41467-017-02500-5 (PMC5792459; doi:10.1038/s41467-017-02500-5)
Supplement: Supplementary file 3 — Description of Additional Supplementary Files(PDF 3 kb) [file 41467_2017_2500_MOESM3_ESM.pdf]

Description of Additional Supplementary Files

File Name: Supplementary Movie 1

Description: 3D visualization of reconstructed nanofibril trajectories in a silk filament shown in Fig. 1j. Fly-through animation of 3D trajectories (cylinders), reconstructed from volume data of z-stack reflectance confocal microscopy images of a sericin-removed silk filament (60× water immersion objective, confocal aperture of 55 µm, stack size of 7.5 µm, and step size of 100 nm), illustrates the high parallelism and volumetric continuity of nanofibrils.
